# Data set on oil palm plantation production and LUC emissions under different management strategies

**DOI:** 10.1016/j.dib.2022.108329

**Published:** 2022-05-29

**Authors:** Jaya Prasanth Rajakal, Raymond R. Tan, Viknesh Andiappan, Yoke Kin Wan, Ming Meng Pang

**Affiliations:** a School of Computer Science & Engineering, Taylor's University, Lakeside Campus, No. 1 Jalan Taylor's, 47500 Subang Jaya, Selangor, Malaysia; b Chemical Engineering Department, De La Salle University, 2401 Taft Avenue, 0922 Manila, Philippines; c Faculty of Engineering, Computing and Science, Swinburne University of Technology, Jalan Simpang Tiga, 93350, Kuching, Sarawak, Malaysia; d Department of Chemical and Environmental Engineering, University of Nottingham Malaysia, Broga Road, 43500 Semenyih, Selangor, Malaysia

**Keywords:** Oil palm plantation, Mathematical model, Optimisation, FFB production, LUC emissions

## Abstract

Oil palm plantations are the fundamental units in a palm supply chain. The fresh fruit bunch (FFB) yield at a plantation varies based on the maturity (age) of the oil palm trees. Failure to account for the maturity can lead to a demand-supply mismatch. To address this issue, Rajakal *et al.* (2021) have developed a mathematical optimisation model to determine the optimal maturity of the plantations needed to meet the crude palm oil demand. This article presents the data set on the FFB production and land use change (LUC) emissions at the plantations. The model was coded and solved in LINGO 18.0. The data can be used for further investigation in optimising other related activities in a palm supply chain.

## Specifications Table

**Table:** 

Subject	Renewable Energy, Sustainability, and Environment.
Specific subject area	Optimal operating state of oil palm plantations
Type of data	Numerical data were obtained from solving the model developed by Rajakal *et al*. (2021). The data is presented in the form of tables and graphs.
How data were acquired	The data were acquired from solving a case study problem to demonstrate the model developed by Rajakal et al. (2021). The model was coded and solved using LINGO 18.0 software in an HP Pavilion x360 laptop with Intel® Core™ i5 8250 (1.80 GHz) processor and 8GB RAM under a 64-bit operating system. The software code of the model is presented in the supplementary material.
Data format	Raw Processed
Parameters for data collection	The parameters required include the cumulative yield profile of the plantations, expansion cost for new plantation development, and land-use change (LUC) emissions.
Description of data collection	The cumulative production data of the plantations were generated from the model, which is described by Rajakal et al. (2021). In this article, the data regarding the annual fresh fruit bunch (FFB) production and annual LUC emissions from the plantations are discussed. Also, the operating capacity required at the palm oil mill and the LUC emissions per ton of FFB production is presented. The raw data for the figures in this article is uploaded in Mendeley data repository and can be accessed in the following link - https://data.mendeley.com/datasets/ms5mc85pwp/3
Data source location	Data presented in this article is collected at Taylor's University, Malaysia.
Data accessibility	The raw data are presented in the Mendeley data repository and can be accessed athttps://data.mendeley.com/datasets/ms5mc85pwp/3 The software code for the case study problem can be accessed at https://data.mendeley.com/datasets/hf9d752t38/1
Related research article	Rajakal, J.P., Tan, R.R., Andiappan, V., Wan, Y.K. and Pang, M.M., 2021. Does age matter? A strategic planning model to optimise perennial crops based on cost and discounted carbon value. Journal of Cleaner Production, 318, p.128526. https://doi.org/10.1016/j.jclepro.2021.128526

## Value of the Data

• The data can be used as benchmark values for LUC emissions per ton of FFB production at the plantations.
• The data can be beneficial to researchers looking at optimisation of oil palm supply chain, life cycle assessment, and carbon management network.
• The data can be used for developing policies on new plantation development.

## 1 Data Description

The data set discussed in this article is processed from the raw data generated from the case study problem solved using the mathematical model developed by Rajakal *et al*. [1]. The case study involves five plantations - P1, P2, P3, P4, and P5. The work intends to determine the optimal maturity required at the plantations based on the expected increase in palm oil demand for ten years planning horizon. The optimal maturity is determined under two scenarios - minimise cost and maximise discounted carbon value (DCV). Besides, the total FFB production and total LUC emission dataset are generated by the model and can be accessed at the Mendeley data repository [2]. This data article presents the dataset on the annual FFB production and annual LUC emissions at each of the plantations for the ten years period.

**Fig. 1:**
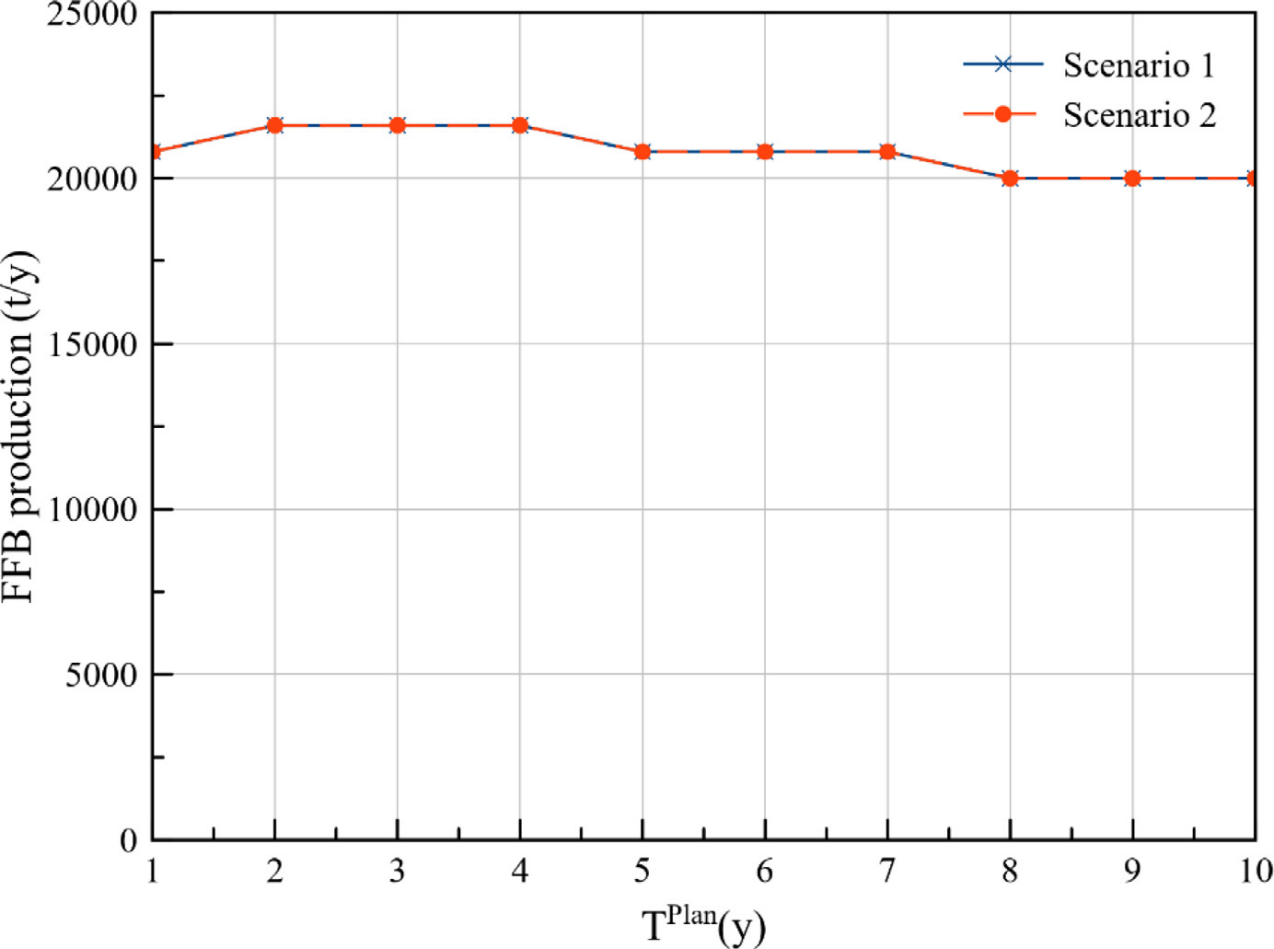
Plantation 1 – FFB production.

**Fig. 2:**
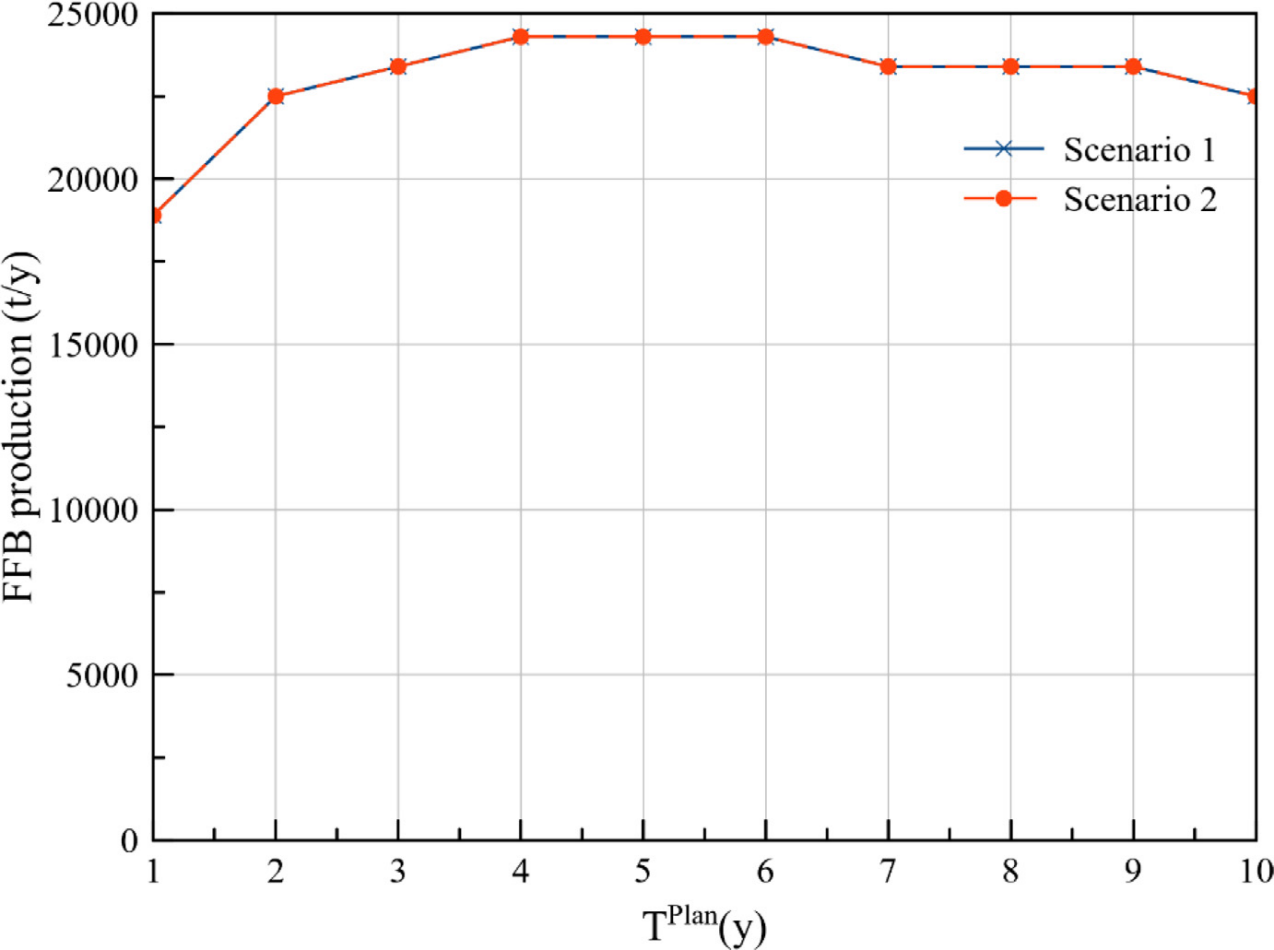
Plantation 2 – FFB production.

**Fig. 3:**
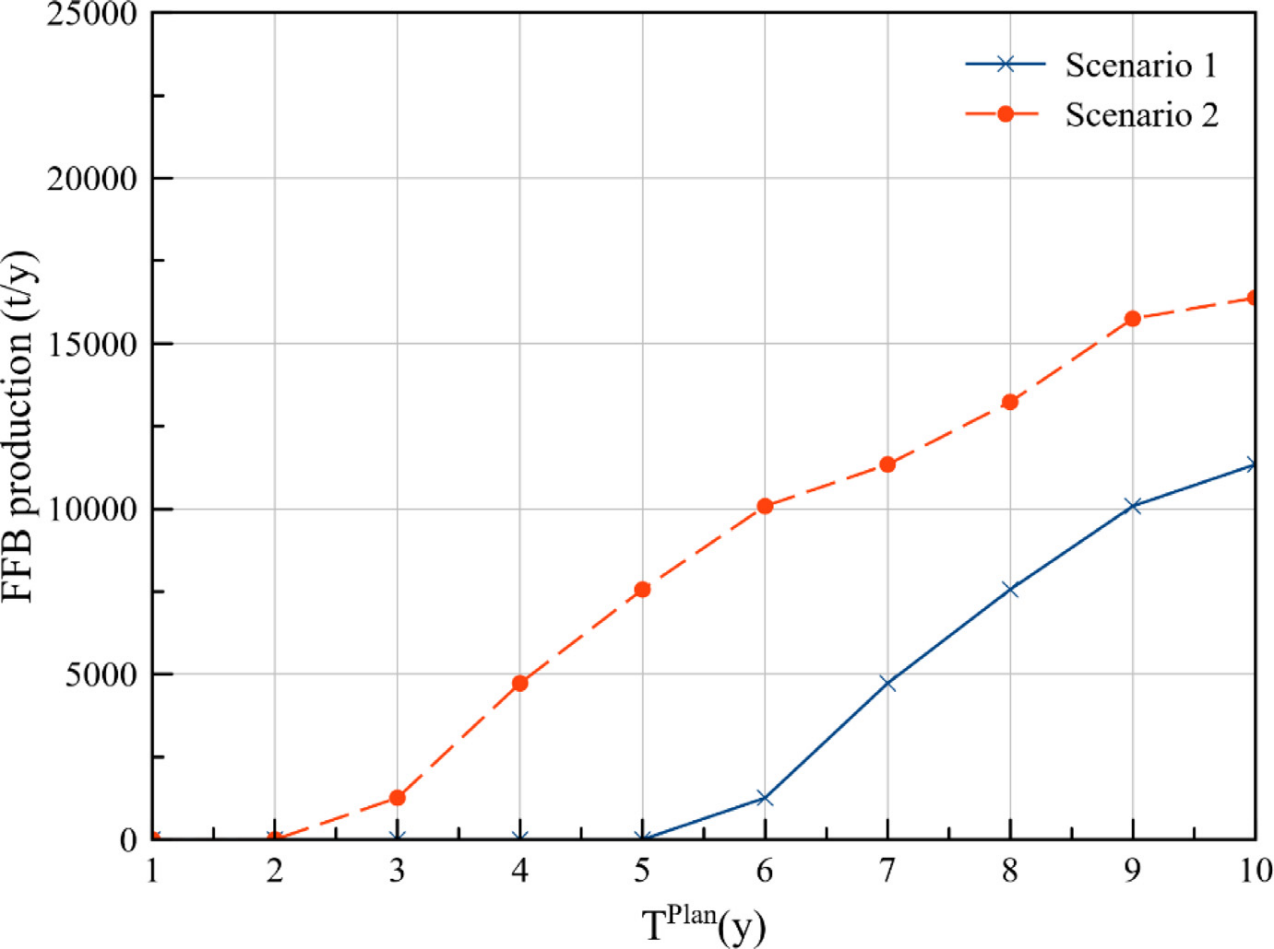
Plantation 3 – FFB production.

**Fig. 4:**
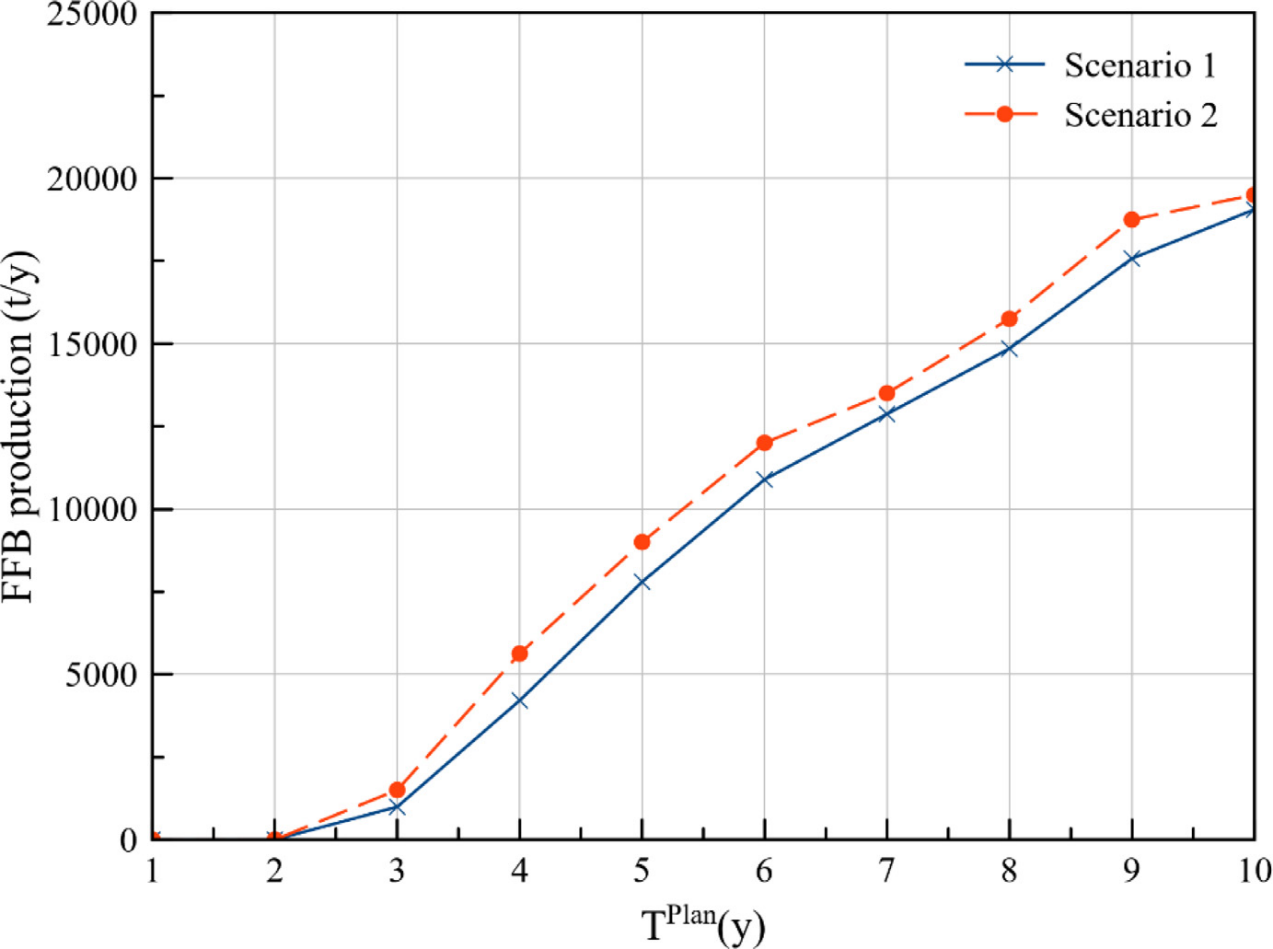
Plantation 4 – FFB production.

**Fig. 5:**
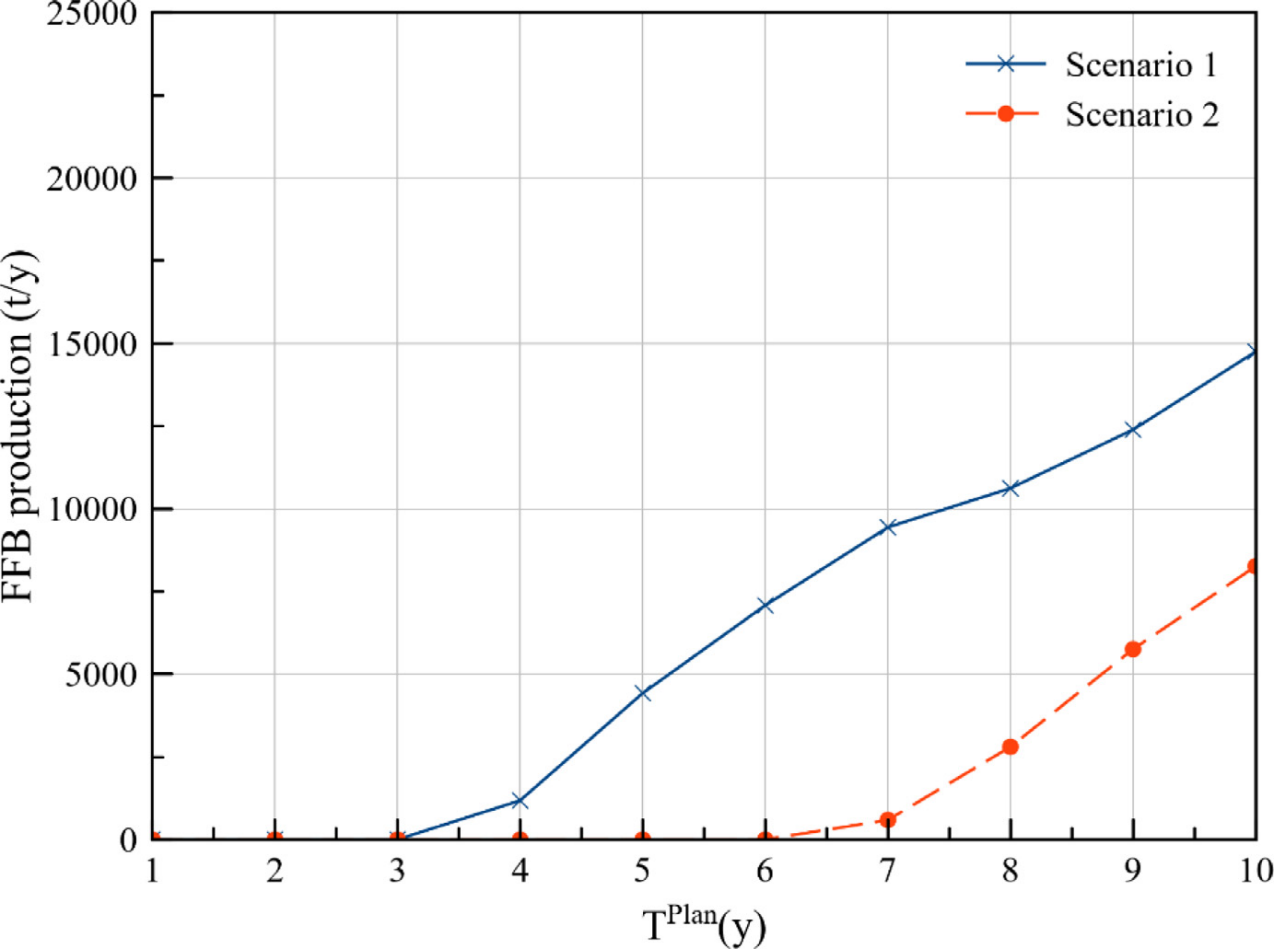
Plantation 5 – FFB production.

**Fig. 6:**
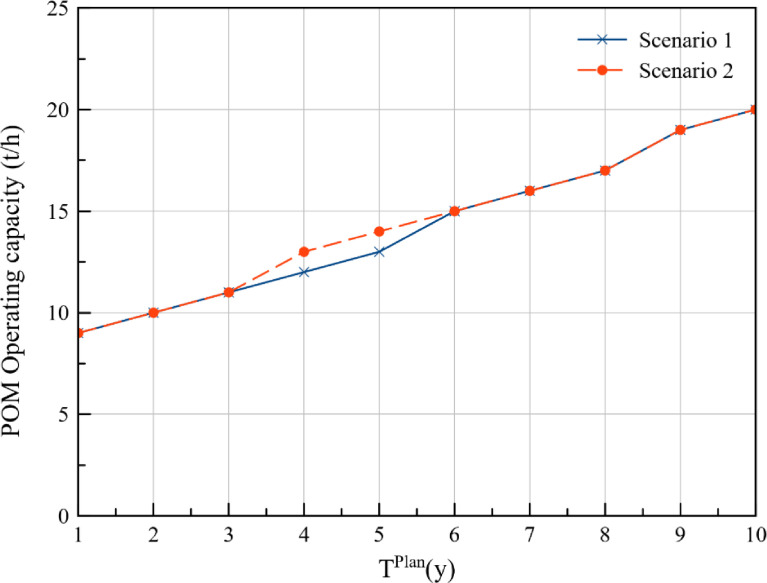
Palm Oil Mill Operating capacity.

The annual FFB production at plantations P1, P2, P3, P4, and P5 for the cost approach and DCV approach is presented in Table 1 and Table 2 respectively. Additionally, comparison of the FFB production between the two optimisation scenarios for each of the plantations is presented in Fig. 1, Fig. 2, Fig. 3, Fig. 4, Fig. 5, while the data points of the figures are provided in the Mendeley data repository. The annual FFB production at P1 and P2 for both the optimisation scenarios are similar as they are existing plantations. However, differences between the scenarios can be observed in P3, P4, and P5 as they are new plantations to be developed at distinct planting periods. The annual FFB production dataset can be valuable for future works on optimising the tactical and operational activities at the plantations. Futhermore, dataset on the operating capacity required at the palm oil mill to process the produced FFBs is presented in Fig. 6.

**Table 1:** Annual FFB production at plantations – Cost approach.

	Annual yield (t/y)				
	Plantation 1	Plantation 2	Plantation 3	Plantation 4	Plantation 5
Year	(800 ha)	(900 ha)	(630 ha)	(750 ha)	(590 ha)
1	20,800	18,900	0	0	0
2	21,600	22,500	0	0	0
3	21,600	23,400	0	450	0
4	21,600	24,300	0	2,738	1,180
5	20,800	24,300	0	6,638	4,425
6	20,800	24,300	1,260	9,900	7,080
7	20,800	23,400	4,725	12,450	9,440
8	20,000	23,400	7,560	14,175	10,620
9	20,000	23,400	10,080	16,650	12,390
10	20,000	22,500	11,340	18,975	14,750

**Table 2:** Annual FFB production at plantations – Discounted carbon value approach.

	Annual yield (t/y)				
	Plantation 1	Plantation 2	Plantation 3	Plantation 4	Plantation 5
Year	(800 ha)	(900 ha)	(630 ha)	(750 ha)	(590 ha)
1	20,800	18,900	0	0	0
2	21,600	22,500	0	0	0
3	21,600	23,400	1,260	1,500	0
4	21,600	24,300	4,725	5,625	0
5	20,800	24,300	7,560	9,000	0
6	20,800	24,300	10,080	12,000	0
7	20,800	23,400	11,340	13,500	0
8	20,000	23,400	13,230	15,750	590
9	20,000	23,400	15,750	18,750	2,803
10	20,000	22,500	16,380	19,500	5,557

**Fig. 7:**
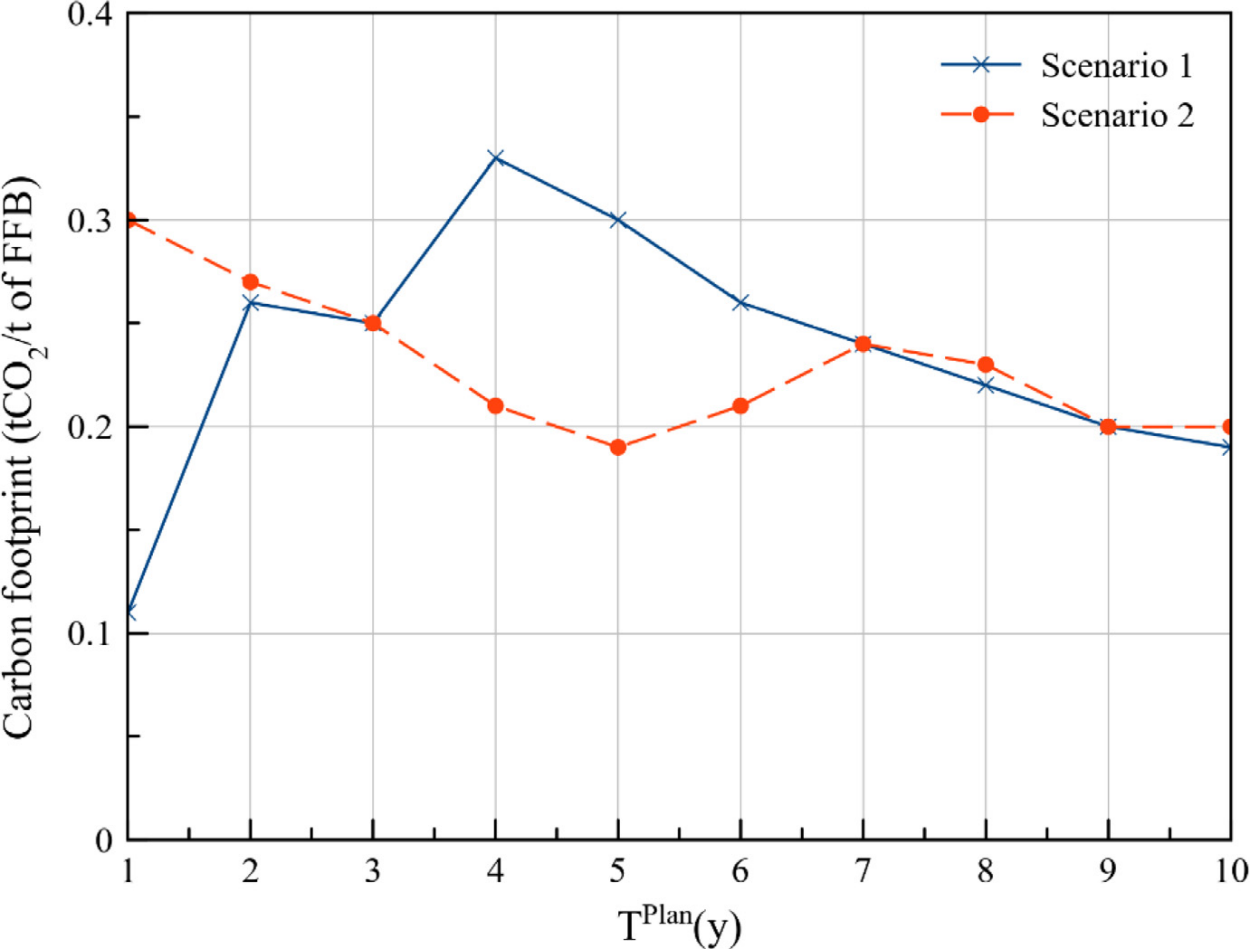
LUC emission per unit FFB production.

The annual LUC emissions from each of the plantations are presented in Table 3 (cost approach) and Table 4 (DCV approach). The LUC emission include direct emissions due to the conversion of tropical forests to oil palm plantations. This emission dataset can used for optimal synthesis of carbon managament networks. Futhermore, the LUC emissions per ton of FFB production (tCO_2_ / t of FFB) for each year is presented in Fig. 7 which can serve as benchmark values for future works to compare with.

**Table 3:** Annual LUC emissions from the plantations – Cost approach.

	LUC emission (tCO_2_)				
Year	Plantation 1 (800 ha)	Plantation 2 (900 ha)	Plantation 3 (630 ha)	Plantation 4 (750 ha)	Plantation 5 (590 ha)
1	Existing Plantations (No land use change)		0	4,606	0
2			0	6,450	5,074
3			0	6,450	5,074
4			5,418	6,450	5,074
5			5,418	6,450	5,074
6			5,418	6,450	5,074
7			5,418	6,450	5,074
8			5,418	6,450	5,074
9			5,418	6,450	5,074
10			5,418	6,450	5,074

**Table 4:** Annual LUC emissions from the plantations – DCV approach.

	LUC emission (tCO_2_)				
Year	Plantation 1 (800 ha)	Plantation 2 (900 ha)	Plantation 3 (630 ha)	Plantation 4 (750 ha)	Plantation 5 (590 ha)
1	Existing Plantations (No land use change)		5,418	6,450	0
2			5,418	6,450	0
3			5,418	6,450	0
4			5,418	6,450	0
5			5,418	6,450	0
6			5,418	6,450	2,397
7			5,418	6,450	5,074
8			5,418	6,450	5,074
9			5,418	6,450	5,074
10			5,418	6,450	5,074

## 2 Experimental Design, Materials and Methods

Rajakal *et al.* (2021) have developed an optimisation model to determine the planting periods for new plantation development based on increasing crude palm oil demand [1]. The work explores the possibility of minimising and delaying peak LUC emissions thereby buying time for climate change mitigation and adaptive measures. The work by Rajakal *et al.,* (2021) is hereafter refered to as “research article” which forms the basis for this data article. The work uses the cumulative yield profile of plantations to determine the optimal planting period under two scenarios – cost approach and discounted carbon value (DCV) approach. The cost approach aims to minimise the LUC emissions while DCV approach which intends to delay the peak LUC emissions. An elaborate discussion on constructing the cumulative yield profile of plantations and the detailed methodology of the optimisation approaches can be found in the research article which can be accessed at https://doi.org/10.1016/j.jclepro.2021.128526.

An illustrative case study problem involving five plantations (P1, P2, P3, P4, and P5) with area of 800 ha, 900 ha, 630 ha, 750 ha, and 590 ha respectively were considered in the research article to demonstrate the developed model. Plantations, P1 and P2 are existing plantations with palm trees having a maturity of 9 y and 7 y while P3, P4, and P5 are potential lands identified for new plantations development. The area and maturity of the plantations are illustrative in defining the problem. Other relevant data required to investigate the case study problem are obtained from secondary sourced like journal publications and government archives.The FFB yield at plantations are considered based on the historical data released by the Malaysian Palm Oil Council (MPOC). The capital cost for new plantation development is estimated from Samad *et al*. [3] and Latif *et al* [4]. Similalry, the operation cost at plantations is taken from Ismail *et al.* [5]. Likewise, the LUC emission values are taken from Agus *et al.* [6].

The optimal planting periods for P3, P4, and P5 are to be determined accounting for the increase in crude palm oil demand for a ten years planning horizon (T^Plan^). The software code for the mathematical model is developed using the equations presented in the research article. The model is coded as a mixed-integer linear programming (MILP) in the LINGO 18.0 software (available for download at www.lindo.com). The software code can be accessed at Mendeley data repository [7]. The software code was solved in an HP Pavilion x360 laptop with Intel® Core™ i5 8250 (1.80 GHz) processor and 8GB RAM under a 64-bit operating system. The processing time taken to achieve the global optimum solution is less than ten seconds. On solving, the optimal planting periods are generated along with data set on total FFB production and total LUC emissions from each of the plantations for the ten years planning horizon. These dataset along with the cumulative yield profile of the plantations form the raw data and can be found in the Mendeley data repository [2]. The results can be reproduced by merely pasting the software codes in the LINGO coding window and then clicking the solve icon in the toolbar.

This data article presents the dataset of the annual FFB production and the annual land use change (LUC) emissions at each of the plantations for each time period (one year) within the planning horizon. The annual FFB production are processed data sets that are deduced from the cumulative yield profile of plantations using the optimal planting periods generated by the model. For example, the optimal planting period determined for P3 under cost approach is 3 years, i.e., third year in the planning horizon. As discussed, cumulative yield profile of P3 is provided in the Mendeley data repository. The annual yield for period *t* is determined by deducing the cumulative yield of period *t* from *t-1*. Table 5 presents the cumulative yield and annual yield of P3 during the considered planning horizon.

**Table 5:** Cumulative and Annual FFB production at P3, 630 ha – Cost approach.

Year	Cumulative yield (t)	Annual yield (t/y)	LUC emissions
1	-	0	0
2	-	0	0
3	0	0	0
*(Planting)*	0	0	0
4	0	0	5418
5	0	0	5418
6	1,260	1,260	5418
7	5,985	4,725	5418
8	13,545	7,560	5418
9	23,625	10,080	5418
10	34,965	11,340	5418

The emission value for the conversion of tropical forests to palm plantations is taken as 8.6 tCO_2_/ha/y from Agus et al. (2013) [6]. The annual LUC emissions are estimated by accounting for the plantation area and the planting period as shown in Table 5. Likewise, the LUC emissions per ton of FFB production (tCO_2_ / t of FFB) for each year is estimated by dividing the annual LUC emissions with the annual FFB production from all the plantations.

## CRediT Author Statement

**Jaya Prasanth Rajakal:** Conceptualisation, Methodology, Data curation, Writing – Original draft preparation, Writing – Reviewing and Editing; **Raymond Tan:** Validation, Writing – Reviewing and Editing; **Viknesh Andiappan;** Conceptualisation, Methodology, Writing – Original draft preparation, Writing – Reviewing and Editing, Supervision; **Yoke Kin Wan:** Conceptualisation, Methodology, Writing – Original draft preparation, Writing – Reviewing and Editing, Supervision. **Pang Ming Meng:** Writing – Reviewing and Editing, Supervision.

## Declaration of Competing Interest

The authors declare that they have no known competing financial interests or personal relationships which have or could be perceived to have influenced the work reported in this article.

## Data Availability

Raw data for annual plantation yield, annual LUC emissions, and mill operating capacity (Original data) (Mendeley Data).
